# A Unified High-Order Semianalytical Model and Numerical Simulation for Bistable Polymer Composite Structures

**DOI:** 10.3390/polym14040818

**Published:** 2022-02-20

**Authors:** Min Sun, Weiliang Gao, Zheng Zhang, Hongcheng Shen, Yisong Zhou, Huaping Wu, Shaofei Jiang

**Affiliations:** 1 College of Mechanical Engineering, Zhejiang University of Technology, Hangzhou 310014, China; sunmin@zjut.edu.cn (M.S.); gweiliang_zjut@163.com (W.G.); shc2632110661@163.com (H.S.); zys596129607@163.com (Y.Z.); hpwu@zjut.edu.cn (H.W.); jsf75@zjut.edu.cn (S.J.); 2 Key Laboratory of Special Purpose Equipment and Advanced Processing Technology, Ministry of Education and Zhejiang Province, Zhejiang University of Technology, Hangzhou 310014, China

**Keywords:** composite structures, bistability, semianalytical model, cross-ply laminate, antisymmetric cylindrical shell

## Abstract

Bistable polymer composite structures are morphing shells that can change shape and maintain two stable configurations. At present, mainly two types of bistable polymer composite structures are being studied: cross-ply laminates and antisymmetric cylindrical shells. This paper proposes a unified semianalytical model based on the extensible deformation assumption and nonlinear theory of plates and shells to predict bistability. Moreover, the higher-order theoretical model is extended for better prediction accuracy, while the number of degrees of freedom is not increased; this ensures a lower computational cost. Finally, based on these theoretical models, the main factors affecting the stable characteristic of the two bistable polymer composite structures are determined by comparing the models of various orders. The main challenges in describing the bistable behavior, such as bifurcation points and the curvatures of stable states, are addressed through prediction of the corner transversal displacement in stable configurations. The results obtained from the theoretical model are validated through nonlinear finite element analysis.

## 1. Introduction

Morphing structures play an important role in the aerospace industry. As a new type of morphing structure, the bistable polymer composite structures have received extensive attention due to their low weight and excellent mechanical properties [1,2]. They have two different configurations, which can both be stable without the need for an ongoing actuation force [3]. Owing to their excellent characteristics and performances, these bistable polymer composite structures have been extensively studied through theoretical models [4,5,6,7,8,9], numerical simulations [10,11,12,13,14], and experiments [15,16,17,18,19]. In addition, research has been conducted in terms of optimization [20,21] and actuation [22,23,24,25,26] of such structures. These structures have also been used in various situations in which morphing and smart structures are required, such as morphing wings [27,28,29,30], energy harvesters [31,32,33,34,35], and bionic structures [36,37,38,39].

**Figure 1:**
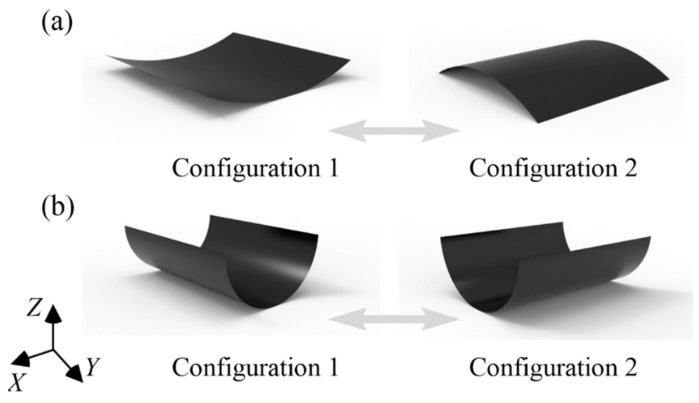
Two different kinds of bistable polymer composite structures: (**a**) CPL; (**b**) ACS.

Based on the manufacturing process, bistable polymer composite structures can be categorized into unsymmetric cross-ply and antisymmetric layups, as shown in Figure 1. The unsymmetric cross-ply laminate (CPL) is obtained by holding and curing at a high temperature and naturally cooling to room temperature [40], whereas the antisymmetric cylindrical shell (ACS) is obtained using a cylindrical steel mold rather than residual thermal stresses [41]. The configurations of the two types of bistable polymer composite structures are relatively regular cylinders. However, the principal curvature directions of the two stable configurations of unsymmetric CPLs are different, whereas those of ACSs are the same.

Furthermore, the theoretical models of the two types of structures, which are employed to predict bistable or multistable configurations and their stable characteristics, are different. The bistability of CPL was first investigated by Hyer [42,43], and the theoretical model is based on an extension of classical lamination theory to account for the bistable behavior. Researchers have continuously improved the theoretical model by increasing the order and number of terms of the displacement polynomial. Higher-order polynomials are used to model the in-plane strain field, and a 14-parameter theoretical model was proposed by Dano et al. [44]. Moreover, a refined high-order theoretical model, up to the 11th order using a complete polynomial as the displacement function of the laminate, was established by Pirrera et al. [45]. In order to reduce the difficulty of solving the high-order model, the model was treated as dimensionless and the path-following numerical method was used to solve it. The high-order model can accurately predict the influence of factors such as aspect ratio and size on the unsymmetric CPL. However, the high order of the set of complete polynomials implied a large computational cost and thus a loss of efficiency of the method.

To improve upon these methods, the snap-through phenomenon and snap loads gradually became the focus of modeling efforts. A simple model for dynamic analysis of the snap-through phenomena was proposed by Diaconu et al. [46]; the model is used to evaluate the initial displacements for the stable states and also to investigate the static and dynamic transitions from one stable state to another. On those grounds, an analytical model was developed by Mukherjee et al. [47], which extends the previously available models to account for the cantilever boundary condition for a special class of hybrid bistable laminates. Seffen [48] applied a simple linear elastic model to ACS with constant curvatures and elliptical planforms. By combining the compatibility condition with the in-plane equilibrium equations, the closed-form solution for the membrane problem can be obtained. The assumption of uniform curvature was extended to linear and quadratic conditions by Vidoli [49]. By simplifying the Föppl–von Kármán (FVK) equations, symmetric boundary conditions are applied while using fewer degrees of freedom. The governing differential equations obtained using the in-plane equilibrium can be solved numerically for ACS with different symmetrical planform shapes. Based on these studies, an accurate and efficient energy-based method is proposed by Lamacchia et al. [50]. The membrane and the bending components of the total strain energy are decoupled using the semi-inverse formulation of the constitutive equations. Transverse displacements are approximated using Lagrange polynomials, and the membrane problem is solved in isolation by combining compatibility conditions and equilibrium equations.

At present, models for predicting the stability of bistable polymer composite structures are usually characterized through a compromise between computational efficiency and accuracy. In this work, a unified high-order model is proposed for two different bistable polymer composite structures. Based on [48,49,50], this model increased the order of the transverse displacement polynomial and could be applied to laminates with symmetrical planform shapes. The stretching strain energy and the bending strain energy in the total potential energy were solved separately to simplify the expression. As with the Rayleigh–Ritz method, the equilibrium configurations of the bistable polymer composite structures were determined via minimization of the total potential energy with respect to the Lagrangian parameters. The stability of the equilibria was then evaluated by assessing the positive definiteness of the system’s Hessian matrix.

The remainder of this paper is organized as follows: In Section 2, the methodology and modeling framework are presented. In Section 3, the processes of numerical simulation of two different bistable polymer composite structures are described, including differences in modeling, application of boundary conditions, and loading methods. In Section 4, the sensitivity analyses of the structures using theoretical and numerical methods are detailed, and the results obtained from the semianalytical method are compared with numerical models developed in ABAQUS. Finally, conclusions are presented in Section 5.

## 2. Semianalytical Model

### 2.1. The Total Potential Energy

The bistable composite laminate was modeled using classical laminate theory combined with the FVK nonlinear hypothesis. The in-plane strains and the out-of-plane displacement were approximated using unknown polynomial functions.

The midplane strains and curvature fields are

(1) $$\begin{array}{l} \varepsilon_{x}^{0}=\frac{\partial u}{\partial x}+\frac{1}{2}{\left(\frac{\partial w}{\partial x}\right)}^{2};\varepsilon_{y}^{0}=\frac{\partial v}{\partial y}+\frac{1}{2}{\left(\frac{\partial w}{\partial y}\right)}^{2};\varepsilon_{xy}^{0}=\frac{1}{2}\left(\frac{\partial v}{\partial x}+\frac{\partial u}{\partial y}+\frac{\partial w}{\partial y}\frac{\partial w}{\partial x}\right)\\ k_{x}=\frac{\partial^{2}w}{\partial x^{2}};k_{y}=\frac{\partial^{2}w}{\partial y^{2}};k_{xy}=2\frac{\partial^{2}w}{\partial x\partial y} \end{array}$$

The compatibility condition related to the midplane strain and curvature can be written as

(2) $$\begin{array}{l} \mathrm{curlcurl}\varepsilon^{0}=k_{x}k_{y}-k_{xy}^{2}=\Delta g\\ \frac{\partial k_{x}}{\partial y}=\frac{\partial k_{xy}}{\partial x};\frac{\partial k_{y}}{\partial x}=\frac{\partial k_{xy}}{\partial y} \end{array}$$

where Δg denotes the variation of the Gaussian curvature with respect to the initial value and curl mean curl operator. For composite laminate, the relationship between the in-plane stress resultant **N**, bending moment resultant **M**, and the midplane strains is defined by the constitutive equation as

(3) $$\left[\begin{array}{c} N\\ M \end{array}\right]=\left[\begin{array}{cc} A & B\\ B & D \end{array}\right]\left[\begin{array}{c} \varepsilon^{0}-e\\ k-h \end{array}\right]$$

where **A**, **B**, and **D** represent the in-plane stretching stiffness matrix, stretching–bending stiffness matrix and bending stiffness matrix, respectively. ***e*** and ***h***, respectively, refer to the initial midplane strain and curvature. By inverting Equation (3),

(4) $$\left[\begin{array}{c} \varepsilon^{0}-e\\ M \end{array}\right]=\left[\begin{array}{cc} A^{*} & B^{*}\\ -B^{*}{}^{T} & D^{*} \end{array}\right]\left[\begin{array}{c} N\\ k-h \end{array}\right]$$

where

(5) $$A^{*}:=A^{-1};B^{*}:=-A^{-1}B;D^{*}:=D-B^{T}A^{-1}B$$

The stable equilibria of the laminate are found as the local minimum of the total potential energy, which is the sum of the stretching and bending energy:

(6) $$U=\frac{1}{2}\int_{-a}^{a}\int_{-b}^{b}\left({\left[\begin{array}{c} \varepsilon^{0}-e\\ k-h \end{array}\right]}^{T}\left[\begin{array}{cc} A & B\\ B & D \end{array}\right]\left[\begin{array}{c} \varepsilon^{0}-e\\ k-h \end{array}\right]\right)dxdy$$

By substituting Equation (4), the energy functional can be transformed in the following form:

(7) $$U=\frac{1}{2}\int_{-a}^{a}\int_{-b}^{b}\left[A^{*}N:N+D^{*}\left(k-h\right):\left(k-h\right)\right]dxdy$$

where the colon operator is the inner product between tensors (summed pairwise product of all elements). To simplify the problem, dimensionless quantities are introduced [49]:

(8) $$\begin{array}{l} X=x/l,Y=y/l,W=w/l,\bar{a}=a/l,\bar{b}=b/l\\ K=R_{0}k,H=R_{0}h\\ \bar{A}=A/A_{11},\;\bar{B}=B/\left(A_{11}R_{0}\right),\bar{D}=D^{*}/D_{11}^{*}\\ \Sigma =N/A_{11},\;\;\;\;\;\;\hat{U}=UR_{0}^{2}/\left(SD_{11}^{*}\right) \end{array}$$

where *S* is the area of laminate, $l=\sqrt{S}$ is the characteristic length used to scale the coordinates for the area of the laminate, and $R_{0}$ is the characteristic radius.

The dimensionless form of the final energy expression is

(9) $$\hat{U}=\frac{1}{2}\int_{-a}^{a}\int_{-b}^{b}\left[\bar{D}\left(K-H\right):\left(K-H\right)+\frac{12R_{0}^{2}}{t_{e}}{\bar{A}}^{-1}\Sigma :\Sigma \right]\;dxdy$$

where $t_{e}=\sqrt{12D_{11}^{*}/A_{11}}$ represents equivalent thickness of the laminate. For transversely isotropic materials, the equivalent thickness is the same as the actual thickness of the laminate, but it is different for orthotropic materials.

### 2.2. Estimation of In-Plane Stress Resultant

In order to calculate the stretching energy for the given transverse displacement more accurately, it was important that the in-plane force equilibrium conditions of the laminate were satisfied. The in-plane force equilibrium equations are written as

(10) $$\begin{array}{l} \nabla \cdot \Sigma =0\;\;\;\;\;\;\;\;\;\;\;\;\;\;\;\;\;\;\;\;\;\;\;\;\;\;\;\mathrm{on}\;\;\;\;\;S\;\\ \Sigma \cdot n=0\;\;\;\;\;\;\;\;\;\;\;\;\;\;\;\;\;\;\;\;\;\;\;\;\;\;\;\;\;\;\mathrm{on}\;\;\;\;\;\partial S\\ \mathrm{curlcurl}\left(A^{-1}\Sigma \right)=\Delta g+\mathrm{curlcurl}\left({\bar{A}}^{-1}\bar{B}K\right) \end{array}$$

where *n* and ∂S respectively represent the outward unit normal at the boundary of the laminate and its domain; $\mathrm{curlcurl}\left({\bar{A}}^{-1}\bar{B}K\right)$ is zero for straight fiber cross-ply laminates and antisymmetric cylindrical shells. Equation (10) is a standard plane-elasticity problem. The closed-form solution can only be obtained in the elliptical domain. If it is a domain of other shapes, such as a rectangle, only numerical solutions can be obtained.

For plane-elasticity problems, the partial differential equation (PDE) can be written as

(11) $$\begin{array}{l} -\nabla \cdot \sigma =f\;\;\;\;\;\;\;\;\;\;\;\mathrm{in}\;\;\Omega \\ \sigma \cdot n=\tau \;\;\;\;\;\;\;\;\;\;\;\;\;\;\;\;\;\;\;\;\;\;\;\;\mathrm{on}\Omega \\ \varepsilon =\frac{1}{2}\left(\nabla u+{\left(\nabla u\right)}^{T}\right),\;\;\;\;\;\;\sigma =\sigma \left(\varepsilon \right) \end{array}$$

where σ is the stress tensor, *f* is the body force per unit volume, and τ is the traction.

To solve Equation (11), it must be transformed into a variational problem. The basic recipe for turning a PDE into a variational problem is to multiply the PDE by a test function *v*, integrate the resulting equation over the domain Ω, and perform integration by parts with second-order derivatives.

After variational processing, Equation (11) becomes

(12) $$\begin{array}{l} -\int_{\Omega }\left(\nabla \cdot \sigma \right)\cdot v\;d\Omega =\int_{\Omega }\sigma :\nabla v\;d\Omega -\int_{\partial \Omega }\left(\sigma \cdot n\right)\cdot vd\Omega =\int_{\Omega }f\cdot v\;d\Omega \\ \int_{\Omega }\sigma :\nabla v\;d\Omega =\int_{\Omega }f\cdot v\;d\Omega +\int_{\partial \Omega }\tau \cdot vd\Omega \end{array}$$

Another feature of variational formulations is that the test function *v* is required to vanish on parts of the boundary; hence, *v* = 0 on the entire boundary ∂Ω. The final variational results of the plane-elasticity problem are

(13) $$\begin{array}{l} a\left(u,v\right)=\int_{\Omega }\sigma :\nabla v\;d\Omega \\ L\left(v\right)=\int_{\Omega }f\cdot v\;d\Omega \end{array}$$

where *a*(*u*, *v*) is known as a bilinear form and *L*(*v*) as a linear form. In order to equate Equation (10) to a standard plane-elasticity problem, the in-plane stress resultant Σ can be written as

(14) $$\Sigma =\Sigma^{a}+\Sigma^{b}$$

By substituting Equation (14) into Equation (10), we obtain

(15) $$\begin{array}{l} -\nabla \cdot \Sigma^{a}=\nabla \cdot \Sigma^{b}\\ \Sigma^{a}\cdot n=-\Sigma^{b}\cdot n \end{array}$$

In comparison with Equation (11), $\Sigma^{a}$ is equivalent to the stress tensor σ; $\nabla \cdot \Sigma^{b}$ is equivalent to the body force *f*; and $-\Sigma^{b}\cdot n$ is equivalent to the traction τ, which can be calculated using a 2×2 symmetric tensor $\Sigma^{b}$ by imposing the condition $\mathrm{curlcurl}\left(A^{-1}\Sigma^{b}\right)=g\left(X,Y\right)$.Therefore, Equation (10) can be completely regarded as a standard plane-elasticity problem to be solved.

### 2.3. High-Order Varying Curvatures (HVCs)

Based on the assumption of uniform curvature, high-order varying curvatures are considered, assuming that the transverse displacement is

(16) $$W=\frac{1}{2}q_{1}X^{2}+\frac{1}{2}q_{2}Y^{2}+q_{3}XY+\frac{1}{\left(n+2\right)\left(n+1\right)}q_{4}X^{n+2}+\frac{1}{\left(n+2\right)\left(n+1\right)}q_{5}Y^{n+2}$$

The unknowns $q_{1},q_{2},q_{3},q_{4},q_{5}$ are the Lagrangian parameters of the model. Then, the associated curvature field is

(17) $$k=\left[\begin{array}{cc} k_{x} & k_{xy}\\ k_{xy} & k_{y} \end{array}\right]=\left[\begin{array}{cc} q_{1}+q_{4}X^{n} & q_{3}\\ q_{3} & q_{2}+q_{5}Y^{n} \end{array}\right]$$

Then, the average curvature of the laminate can be written as

(18) $$\begin{array}{l} {\bar{k}}_{x}=\frac{\int_{S}k_{x}dS}{S}=q_{1}+\frac{\left({\left(-\bar{a}\right)}^{n}+{\bar{a}}^{n}\right)q_{4}}{2\left(1+n\right)};\\ {\bar{k}}_{y}=\frac{\int_{S}k_{y}dS}{S}=q_{2}+\frac{\left({\left(-\bar{b}\right)}^{n}+{\bar{b}}^{n}\right)q_{5}}{2\left(1+n\right)};\\ {\bar{k}}_{xy}=\frac{\int_{S}k_{xy}dS}{S}=q_{3} \end{array}$$

The variation of the Gaussian curvature can be obtained by Equation (2):

(19) $$\begin{array}{l} \Delta g=\lambda_{1}+\lambda_{2}X^{n}+\lambda_{3}Y^{n}+\lambda_{4}X^{n}Y^{n}\\ \lambda_{1}=q_{1}q_{2}-q_{3}^{2}-g_{H};\lambda_{2}=q_{2}q_{4};\lambda_{3}=q_{1}q_{5};\lambda_{4}=q_{4}q_{5} \end{array}$$

For the HVC polynomial, it is necessary to solve four PDE problems:

(20) $$\begin{array}{l} \nabla \cdot \Sigma_{i}^{a}=0\;\;\;\;\;\;\;\;\;\;\;\;\;\;\;\;\;\;\;\;\;\;\;\;\;\;\;\mathrm{on}\;\;\;\;\;S\;\\ \Sigma_{i}^{a}\cdot n=0\;\;\;\;\;\;\;\;\;\;\;\;\;\;\;\;\;\;\;\;\;\;\;\;\;\;\;\;\;\;\mathrm{on}\;\;\;\;\;\partial S\\ \mathrm{curlcurl}\left(A^{-1}\Sigma_{i}^{a}\right)=\Delta g_{i} \end{array}$$

where for $i=1,2,3,4,\;\;\;\Delta g_{i}=1,X^{n},Y^{n},X^{n}Y^{n}$, respectively. The in-plane stress field is approximated as

(21) $$\Sigma =\sum_{i=1}^{4}\frac{l^{2}}{R_{0}^{2}}\left(\lambda_{i}\Sigma_{i}^{a}\right)$$

The stretching energy equation in Equation (9) can be modified as follows:

(22) $${\hat{U}}_{s}=\frac{1}{2}\sum_{i=1}^{4}\sum_{j=1}^{4}\lambda_{ij}\Psi_{ij}$$

Furthermore, the total energy equation is modified as follows:

(23) $$\hat{U}=\frac{1}{2}\int_{S}\left[\bar{D}\left(K-H\right):\left(K-H\right)\right]\;dS+\frac{1}{2}\sum_{i=1}^{4}\sum_{j=1}^{4}\lambda_{ij}\Psi_{ij}$$

where $\lambda_{ij}=\lambda_{i}\lambda_{j};\Psi_{ij}=\int_{S}{\bar{A}}^{-1}\Sigma_{i}^{a}:\Sigma_{j}^{a}dS\;\;\;\;\;\;\left(i,j=1,2,3,4\right)$. It is crucial to solve the stretching–bending factor $\Psi_{ij}$, because it determines the relative weight of bending and stretching energies in Equation (23).

Equilibrium configurations correspond to extrema of $\Psi_{ij}$, therefore satisfying the expression

(24) $$f_{i}\;\;=\frac{\partial \hat{U}}{\partial q_{i}}=0\;\;\;\;\;\;\;\;\;\;\;\;\;\;(i=1,2,3,4,5)$$

which results in a set of nonlinear equilibrium equations of the kind $f_{i}\;\;=0$. The stability of the solutions of Equation (24) is assessed by confirming the positive definiteness of the Hessian matrix of the total potential energy. Equilibria are stable if and only if

(25) $$\frac{\partial^{2}\hat{U}}{\partial^{2}q_{i}}>0\;\;\;\;\;\;\;\;\;\;\;\;(i=1,2,3,4,5)$$

### 2.4. Selection of $\Sigma^{b}$

For the rectangular domain, these PDE problems cannot be solved in the closed form. Therefore, a standard finite element method was used for their numerical solution as part of the FEniCS project. To select the most suitable $\Sigma^{b}$ and find the value of $\Sigma_{i}^{a}$, $\mathrm{curlcurl}\left(A^{-1}\Sigma^{b}\right)=g\left(X,Y\right)=1$ is considered as an example to discuss.

(1) Consider one item in the $\Sigma^{b}$:

(26) $$\Sigma_{11}^{b}=\left[\begin{array}{cc} Y^{2}/2 & 0\\ 0 & 0 \end{array}\right],\Sigma_{12}^{b}=\left[\begin{array}{cc} 0 & 0\\ 0 & X^{2}/2 \end{array}\right],\Sigma_{13}^{b}=\left[\begin{array}{cc} 0 & -XY/2\\ -XY/2 & 0 \end{array}\right]$$

(2) Consider two items in the $\Sigma^{b}$:

(27) $$\Sigma_{21}^{b}=\left[\begin{array}{cc} Y^{2}/4 & 0\\ 0 & X^{2}/4 \end{array}\right],\Sigma_{22}^{b}=\left[\begin{array}{cc} 0 & -XY/4\\ -XY/4 & X^{2}/4 \end{array}\right],\Sigma_{23}^{b}=\left[\begin{array}{cc} Y^{2}/4 & -XY/4\\ -XY/4 & 0 \end{array}\right]$$

(3) Consider three items in the $\Sigma^{b}$:

(28) $$\Sigma_{31}^{b}=\left[\begin{array}{cc} Y^{2}/8 & -XY/4\\ -XY/4 & X^{2}/8 \end{array}\right]$$

**Figure 2:**
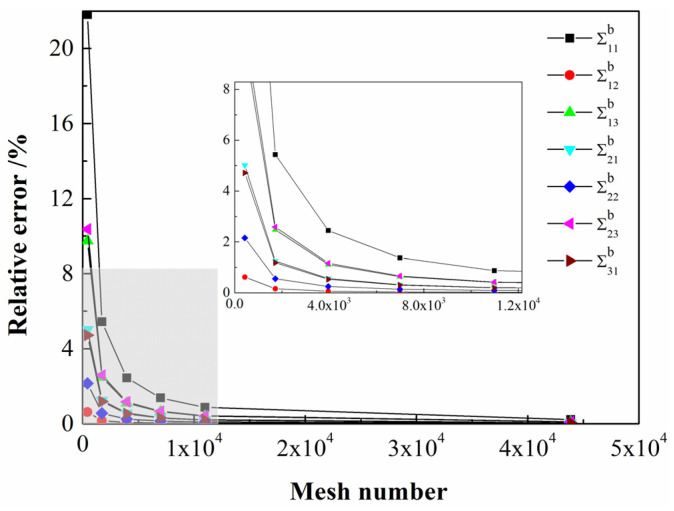
The relative error between the numerical solution and the closed form of stretching–bending factor $\Psi_{ij}$.

The closed-form solution of the elliptical domain has been given in [48]. When considering the selection of different $\Sigma^{b}$ in the FEniCS project, the variation of $\Psi_{ij}$ with the number of mesh could be obtained. Thus, the relative error between the numerical solution and the closed form could be obtained, as shown in Figure 2. As the number of mesh increases, choosing various $\Sigma^{b}$ generates a sufficiently accurate value of $\Psi_{ij}$. However, it can be seen from Figure 2 that the relative error calculated by selecting $\Sigma_{12}^{b}$ under the same number of mesh is the smallest. Therefore, consider choosing $\Sigma_{12}^{b}$ to calculate the value of $\Psi_{ij}$.

## 3. Finite Element Simulation

**Figure 3:**
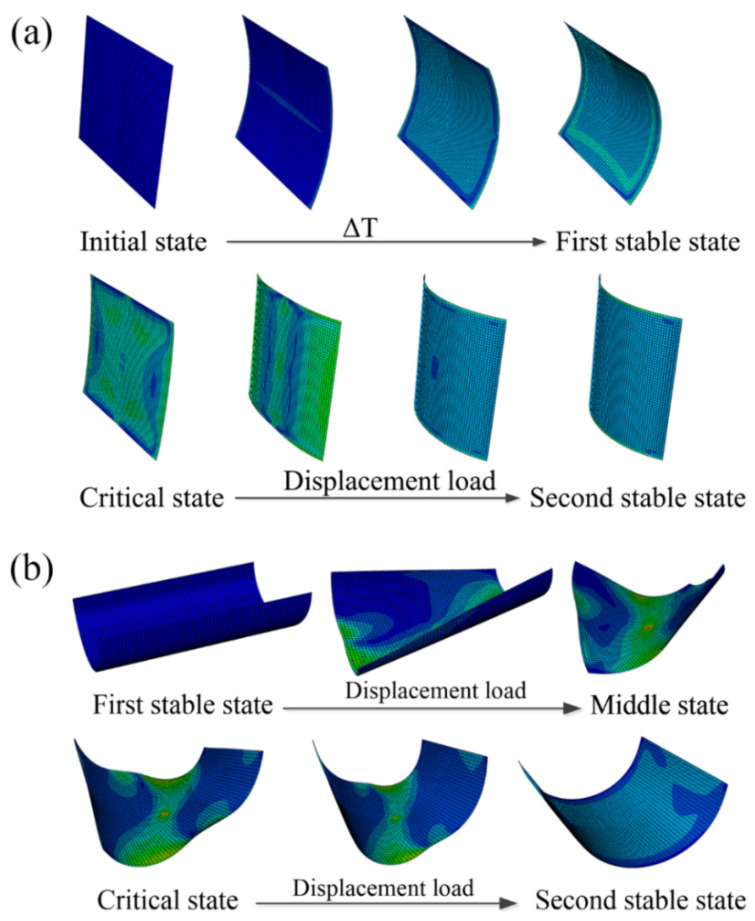
Simulated morphing process of bistable polymer composite structures: (**a**) CPL; (**b**) ACS.

Although the analytical model can predict stable states conveniently, validation based on finite element analysis (FEA) is still required to confirm the accuracy of the predictions. However, even in this validation stage, the analytical model plays a key role in finding different stable configurations. The material properties used in the numerical simulation are listed in Table 1. The nonlinear finite element software ABAQUS was used to simulate the morphing processes of the bistable polymer composite structures, as shown in Figure 3. For the two different types of bistable polymer composite structures, the simulation process varied.

### 3.1. Simulation of CPL

**Figure 4:**
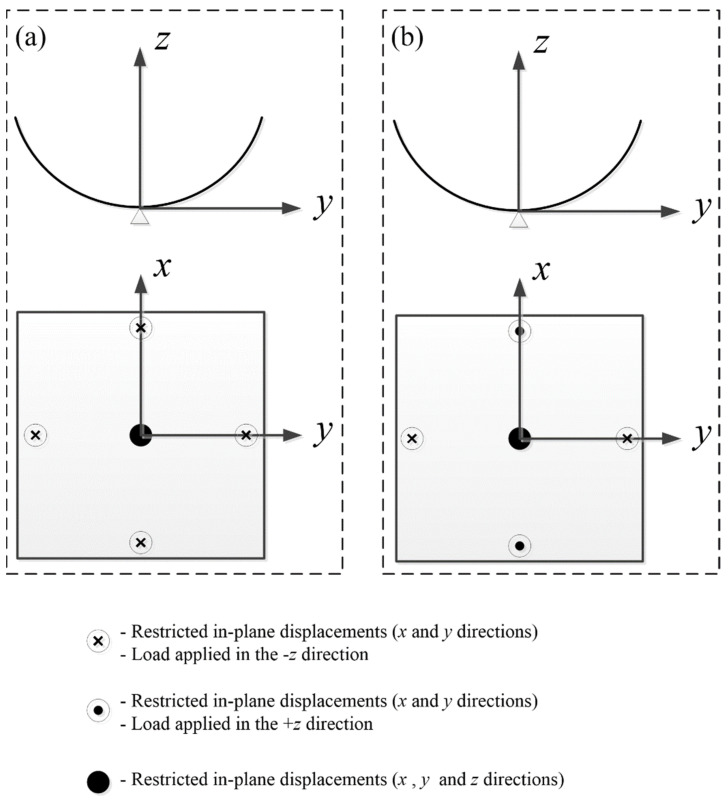
Boundary condition setting in the simulation process of bistable polymer composite structures: (**a**) CPL; (**b**) ACS.

Reduced integration can provide more accurate results and significantly reduced computational time. The S4R reduced integration shell element is chosen here for its better convergence. The FEA process for the CPL includes four steps: (1) Modeling process: The CPL was modeled with a planar shell (3D deformable shell), and the stacking sequence was [0/90]. (2) Curing process: An initial temperature field of 140 °C was applied to the laminate and was then reduced to 20 °C to obtain the first stable state. (3) Loading process: To obtain another stable state, as shown in Figure 4a, the center node of the laminate was fully constrained, and four displacement loads in the same direction were applied on the midpoints of the four boundaries. The options were set as “Nlgeom”, and stabilization with dissipated energy fraction was utilized as a default parameter. (4) Unloading process: We deactivated the displacement loads to obtain the second stable state with the option Nlgeom still on and stabilize off to avoid inaccuracies.

In the postprocessing, the output of the curvatures of the second stable state of the laminate was the average of the curvatures of all shell elements, as in the theoretical assumption. The results of FE simulations for all specimens are discussed in Section 4.

### 3.2. Simulation of ACS

Compared with the simulation process of CPL, the curing process is not included in the simulation of the ACS, and the first stable state after the curing process was directly given following the modeling process. As the curing process of ACS was completed using a cylindrical steel mold, the radius of curvature of the first stable state was known and approximately equal to the radius of the mold. In the modeling process, the stacking sequence of the ACS was set as [45/−45/45/−45]. As shown in Figure 4b, similar to the CPL, the center node of the shell was also constrained, and two pairs of displacement loads in different directions were applied on the midpoints of the four boundaries.

## 4. Results and Discussion

The multiple parameters that affect the stable state characteristics of bistable polymer composite structures are discussed in this section. The main parameters that affect the bistable characteristics of CPL are temperature variation ΔT, ply thickness *t*, aspect ratio *a*/*b*, and side lengths *a* and *b*. For ACS, central angle γ, longitudinal length L=2a, ply angle α, and initial curvature $h_{0}$ are the main design parameters. Therefore, it is important to predict the bifurcation point under various sensitivity factors. The order of the transverse displacement polynomial (Equation (16)) was progressively increased until the desired accuracy was achieved. It must be emphasized that numerical accuracy with respect to FEA was not considered a primary goal. The curvature field (Equation (17)) was truncated at orders *n* = 2, 4, 6, 8. Most of the numerical simulations presented herein converged at order 6 (Ord. 6).

### 4.1. CPL

**Figure 5:**
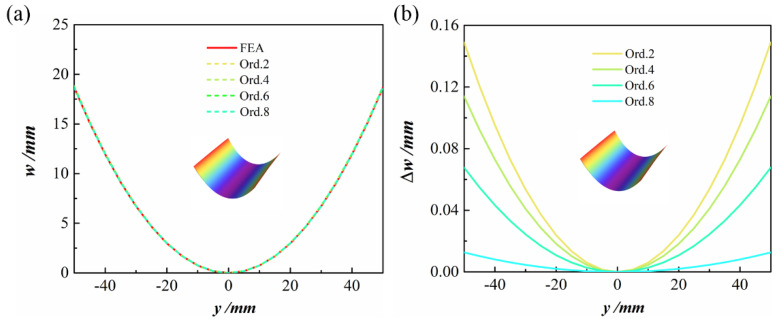
The cross-section configuration of the second stable state of CPL: (**a**) out-of-plane displacement at *x* = 0 of the second stable state; (**b**) differences between various orders and FE out-of-plane displacement results.

To highlight the difference between various orders and the FEA results more intuitively, Figure 5 shows the cross-section configuration of the second stable state of 100 mm×100 mm, [0/90] CPL. It can be seen from Figure 5a that increasing the order does not lead to a major difference in the configuration diagram. Moreover, as shown the Figure 5b, the maximum error between the theoretical model and the FE result is at the edge. The maximum errors between the theoretical results of Ord. 2, Ord. 4, Ord. 6, and Ord. 8 and those of the finite element simulation were 0.149, 0.114, 0.068, and 0.013 mm, respectively. The relative error was reduced from 0.797% for Ord. 2 to 0.068% for Ord. 8. The prediction accuracy of the model increased with the order, but the number of degrees of freedom did not increase. The computational time did not increase either.

#### 4.1.1. Temperature Variation

**Figure 6:**
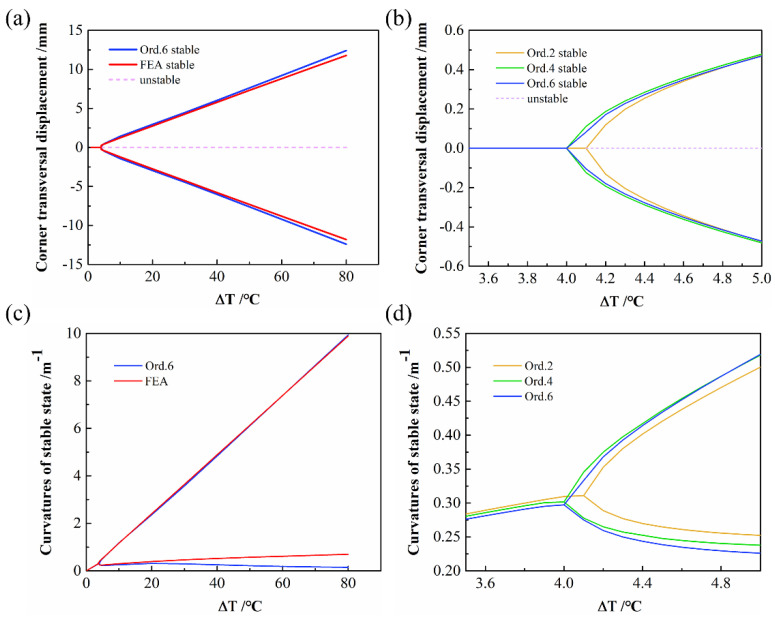
Bifurcation diagram with temperature variation: (**a**) transversal displacement of the CPL corners against the temperature variation; (**b**) close-up of bifurcation point of transversal displacement; (**c**) curvatures of stable state against the temperature variation; (**d**) close-up of bifurcation point of curvatures of stable state.

During the curing process, the CPL is cooled from the curing temperature to room temperature and experiences inelastic deformation caused by the thermal effect, which leads to warping. Then, CPL exhibits two stable configurations. As shown in Figure 6, when keeping the size and thickness of the CPL constant, the bifurcation phenomenon occurs with a continuous increase in the curing temperature differences, which explains the existence of bistable behavior.

The FE results are represented by the red line in Figure 6a; the solid line and the dashed line represent stable and unstable configurations, respectively. To compare the prediction results for the bifurcation points of various orders, a close-up of the bifurcation points of orders 2, 4, and 6 is shown in Figure 6b. It can be seen that the predicted bifurcation point of Ord. 2 is 4.1 °C, whereas the curves of Ord. 4 and Ord. 6 are essentially coincident, and the predicted bifurcation points are both 4 °C. From Ord. 2 to Ord. 6, a difference of 0.1 °C is observed; moreover, as the order increases, the bifurcation point moves toward the convergence direction. The curvatures of the stable state with temperature are shown in Figure 6c,d. The bifurcation points predicted for various orders can be obtained similarly.

#### 4.1.2. Ply Thickness

**Figure 7:**
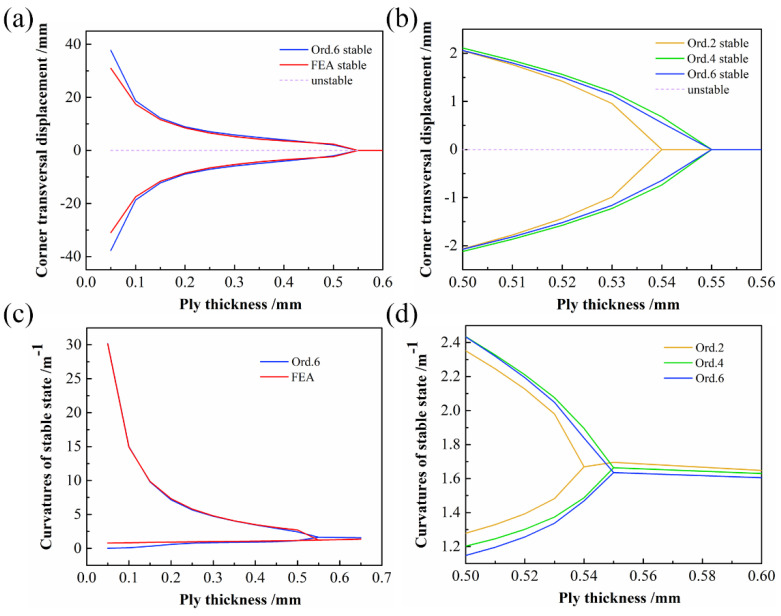
Bifurcation diagram with varying ply thickness: (**a**) transversal displacement of the CPL corners against the ply thickness; (**b**) close-up of bifurcation point of transversal displacement; (**c**) curvatures of stable state against the ply thickness; (**d**) close-up of bifurcation point of curvatures of stable state.

In the next analysis, we maintained other parameters constant and changed the ply thickness of the CPL to obtain the relationships between corner transversal displacement, curvatures of the stable state, and ply thickness, as shown in Figure 7. When the CPL is in a stable configuration, the corner transversal displacement and curvature of the stable state decrease nonlinearly with the increase in the ply thickness, and bifurcation occurs. From Ord. 2 to Ord. 6, the prediction values of the thickness bifurcation point were 0.54, 0.55, and 0.55 mm, respectively.

#### 4.1.3. Aspect Ratio

**Figure 8:**
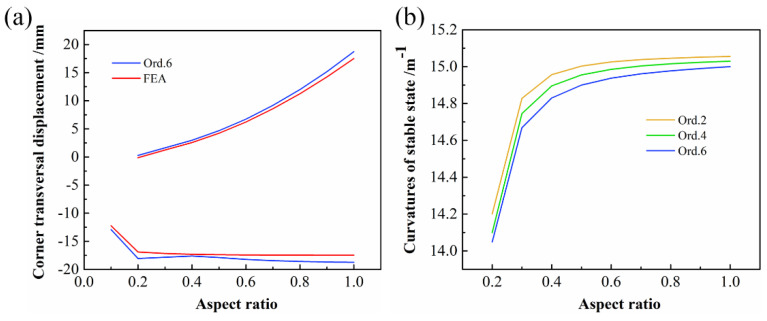
Influence diagram of aspect ratio: (**a**) transversal displacement of the CPL corners against the aspect ratio; (**b**) curvatures of stable state against the aspect ratio.

The effect of the aspect ratio on the stable configuration of CPL is shown in Figure 8. Side length *b* was held constant, and the aspect ratio was changed by changing the value of side length *a.* As the aspect ratio decreased, the solutions reached a turning point and the plate lost its bistability. The transversal displacement of the corner points is less affected by the width of the laminate when the CPL has two stable configurations and bends along the length. Figure 8b shows the relationship between the curvature of the second stable state and the aspect ratio. Curves of all orders were within a similar range of aspect ratio, showing an increasing trend and then converging to a certain value.

#### 4.1.4. Side Length

**Figure 9:**
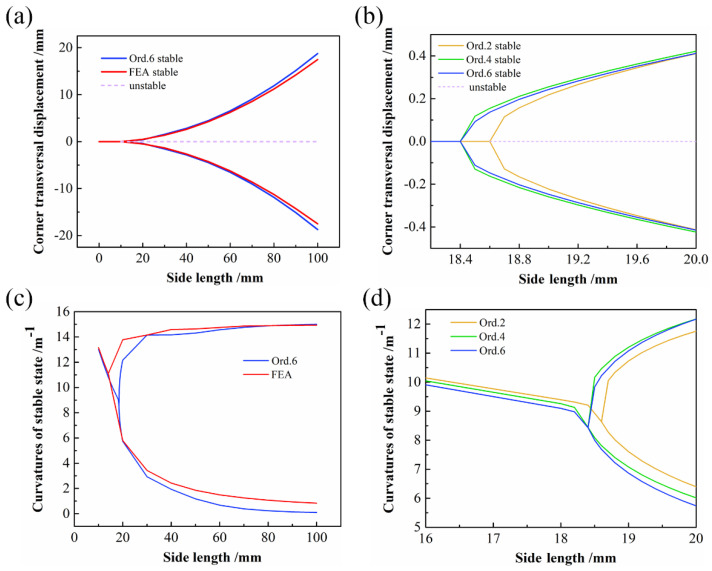
Bifurcation diagram of varying side length: (**a**) transversal displacement of the CPL corners against the side length; (**b**) close-up of bifurcation point of transversal displacement; (**c**) curvatures of stable state against the side length; (**d**) close-up of bifurcation point of curvatures of stable state.

In order to explore the influence of side length on the stable configuration of CPL, the aspect ratio of CPL was kept at 1. As shown in Figure 9, as the side length increases, the bifurcation phenomenon reappears. The predicted bifurcation point of Ord. 2 was 18.6 mm, whereas the Ord. 4 and Ord. 6 points were both 18.4 mm. Moreover, as the order increased, the bifurcation point once again converged to Ord. 6. Figure 9c,d shows that the overall trends of curvature prediction with various order assumptions are approximately the same.

### 4.2. ACS

**Figure 10:**
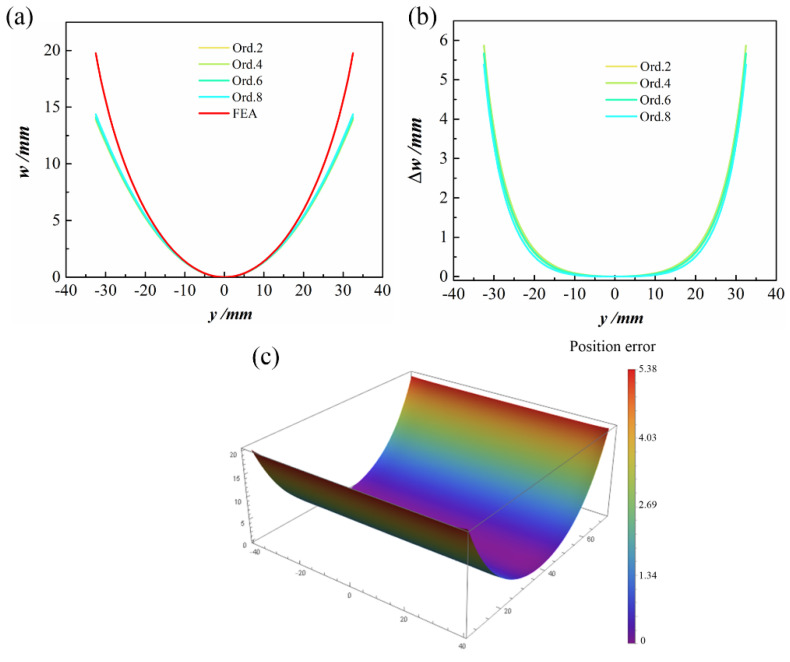
The cross-section configuration of second stable state of ACS: (**a**) out-of-plane displacement at *x* = 0 of the second stable state; (**b**) differences with respect to FEA; (**c**) position error between predictions at Ord. 8 and FEA.

The geometric parameters of the ACS were as follows: central angle γ=180°, longitudinal length *L =* 80 mm, stacking sequence [45/−45/45/−45], and initial curvature $h_{0}=40\;m^{-1}$. Figure 10a,b shows the out-of-plane displacement at *x* = 0 of the second stable state and the differences between theoretical predictions at various orders and the FE simulation. The prediction accuracy of the theoretical model on the edge of the shell is relatively low, though the maximum error did not exceed 6 mm. A comparison with respect to FE results is presented in Figure 10c, and the color map superimposed on the structure represents the difference between the FE and theoretical solutions under the Ord. 8 assumption.

#### 4.2.1. Ply Angle

**Figure 11:**
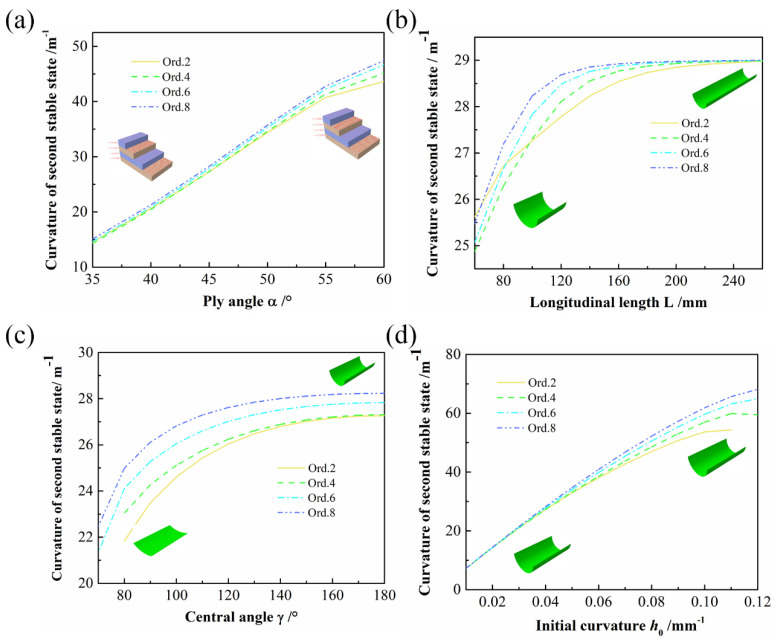
The influence of various parameters on the curvature of the second stable state of ACS: (**a**) ply angle; (**b**) longitudinal length; (**c**) central angle; (**d**) initial curvature.

As the first stable state of ACS is determined by the mold, only the variation of the curvature of the second stable state is discussed here. As shown in Figure 11a, the curves of various orders predicting the influence of the ply angle on the curvature of the second stable state are essentially coincident. When the ply angle was relatively large, the prediction results from Ord. 2 to Ord. 8 showed that the higher the order was, the larger the curvature of the second stable state became. However, the maximum relative error between the minimum value of Ord. 2 and the maximum value of Ord. 8 did not exceed 8%. All showed a gradually increasing trend of curvature of the second stable state and demonstrated that the ply angle had a significant influence on the bistability of ACS.

#### 4.2.2. Longitudinal Length

Figure 11b presents the relationship between the longitudinal length of the ACS and the curvature of the second stable state. As the longitudinal length gradually increases, the predicted results from Ord. 2 to Ord. 8 converge to 29 m^−1^, and as the order increases, the curves converge faster. Owing to the gradual increase in longitudinal length, the second stable configuration of the ACS will become a rolled cylindrical shape. If the longitudinal length was increased on this basis, the curvature of the second stable state would remain basically constant.

#### 4.2.3. Central Angle

The central angle is another important design parameter of the ACS, in addition to the curvature of the second stable state against the central angle, as shown in Figure 11c. All show a slowly increasing trend of curvature of the second stable state. Moreover, as the central angle increases, the curvature of the second stable state gradually converges. It can be seen from Figure 11c that the convergence values of Ord. 2 and Ord. 4 are both 27.3 m^−1^, whereas those of Ord. 6 and Ord. 8 are 27.8 and 28.2 m^−1^, respectively.

#### 4.2.4. Initial Curvature

The influence of the initial curvature on the curvature of the second stable state is shown in Figure 11d. The curves in the figure basically coincide when the initial curvature is small, and all of them show that the curvature of the second stable state increases with the increase in the initial curvature.

## 5. Conclusions

There are two main types of bistable polymer composite structures: CPL and ACS. The theoretical models predicting their bistable behavior are not uniform. In this paper, an efficient and accurate unified semianalytical model HVC was proposed. The model was based on the uniform curvature assumption and extended to the nonuniform curvature. In addition, the order of the curvature polynomial was increased to the eighth order. Through a CPL and ACS case study, it was shown that, despite bistable characteristics being well resolved already at the second order, other aspects of the nonlinear behavior of the bistable composite structure were only captured at higher orders. By combining the semianalytical model with the numerical method, the parameters influencing CPL and ACS could be discussed systematically. The temperature variation, ply thickness, aspect ratio, and side length of CPL and the ply angle, longitudinal length, central angle, and initial curvature of ACS were analyzed in detail. In principle, the order of curvature polynomial could be increased indefinitely. However, the prediction of the bifurcation point in CPL for the fourth and sixth orders reached satisfactory results. For ACS, the influence of each parameter under various orders on the curvature of the second stable state was basically the same. In the future, higher-order or more complex approximation polynomials might be considered for the curvature field to enhance the predictive capabilities of the model.

## Figures and Tables

**Table 1:** Material properties of T700/epoxy unidirectional lamina.

Lamina Properties	Value	Units
Longitudinal Young’s modulus (*E*_1_)	120	GPa
Transverse Young’s modulus (*E*_2_)	8.42	GPa
Poisson’s ratio (*ν*_12_)	0.25	
Shear modulus (*G*_12_)	4.5	GPa
Longitudinal thermal expansion coefficient (*α*_1_)	−3.01 × 10^−6^	°C^−1^
Transverse thermal expansion coefficient (*α*_2_)	2.773 × 10^−5^	°C^−1^
Ply thickness (*t*)	0.10	mm
